# Delayed Progression of Ataxia with a Static Cerebellar Lesion– Consider SCA27B

**DOI:** 10.1007/s12311-025-01786-2

**Published:** 2025-01-16

**Authors:** Tsz Hang Wong, Jamie Manuputty, Tom van Seeters, Erik-Jan Kamsteeg, Bart van de Warrenburg

**Affiliations:** 1 Department of Neurology, Medisch Spectrum Twente, Koningstraat 1, Enschede, 7512 KZ The Netherlands; 2 Department of Neurology, Elisabeth-TweeSteden Ziekenhuis, Tilburg, The Netherlands; 3 Department of Radiology, Elisabeth-TweeSteden Ziekenhuis, Tilburg, The Netherlands; 4 Department of Human Genetics, Radboud University Medical Center, Nijmegen, The Netherlands; 5 Department of Neurology, Donders Institute for Brain, Cognition & Behaviour, Radboud University Medical Center, Nijmegen, The Netherlands

**Keywords:** SCA27B, *FGF14* mutation, Cerebellar ataxia, Downbeat nystagmus

## Abstract

Repeat expansions in the fibroblast growth factor 14 gene (*FGF14*), associated with spinocerebellar ataxia type 27B (SCA27B), have emerged as a prevalent cause of previously unexplained late-onset cerebellar ataxia. Here, we present a patient with residual symptom of gait ataxia after complicated meningioma surgery, who presented with progressive symptoms of oculomotor disturbances, speech difficulties, vertigo and worsening of gait imbalance, twelve years post-resection. Neuroimaging revealed a surgical resection cavity in the dorsolateral side of the left cerebellar hemisphere, accompanied by gliosis in left cerebellar hemisphere extending into the vermis, extensive non-specific supratentorial periventricular white matter abnormalities, and mild atrophy of the cerebellar vermis. Initially, her symptoms were attributed to re-emergence of her cerebellar symptoms related to the static cerebellar lesion, and due to a failure of compensatory mechanisms with aging. However, the progressive nature of her cerebellar symptoms and the emergence of novel downbeat nystagmus prompted genetic testing for *FGF14* repeat expansion, confirming SCA27B as a significant contributor to her delayed, progressive cerebellar symptoms. This case highlights the significance of considering SCA27B in the differential diagnosis of delayed progressive cerebellar ataxia with oculomotor abnormalities in the presence of a static cerebellar lesion.

## Introduction

Sporadic, late-onset cerebellar ataxia (LOCA) has a wide differential diagnosis, encompassing various acquired, genetic, and non-genetic degenerative diseases. In many cases, the exact cause remains unknown, and the condition is then referred to as an idiopathic, likely degenerative LOCA. The recent discovery of intronic GAA-repeat expansions in the fibroblast growth factor 14 gene (*FGF14*) has shown that a relevant portion of patients with previously unexplained LOCA carries such an expansion (ATX-*FGF14*; spinocerebellar ataxia 27B) [1]. Despite its recent discovery, accumulating evidence indicates that SCA27B is one of the most prevalent causes of adult-onset cerebellar ataxia [1, 2]. Establishing a diagnosis of SCA27B is of significant importance, as treatment with 4-aminopyridine has demonstrated symptomatic benefits for ataxic symptoms and oculomotor disturbances [2].

We here present a case that illustrates that also delayed progression of cerebellar ataxia that emerges in the setting of a known static cerebellar lesion warrants genetic testing of *FGF14*.

## Case Presentation

A 71-year-old woman was referred to our outpatient clinic for a second opinion because of progressive cerebellar ataxia. Her medical history included depression and a cerebellar meningioma resection at age 55. At the time, the procedure was complicated by post-surgical cerebellar edema leading to cerebellar ataxia, which was managed by decompression of the posterior fossa. Post-surgery, she complained about diplopia, dysarthria and limb coordination difficulties. Neurological examination showed mild weakness and impaired coordination of the left arm and leg, horizontal diplopia on left and right lateral gaze, and gait ataxia. She was referred for rehabilitation, and the clinical picture later improved to a mild gait ataxia as the main residual symptom.

**Fig. 1:**
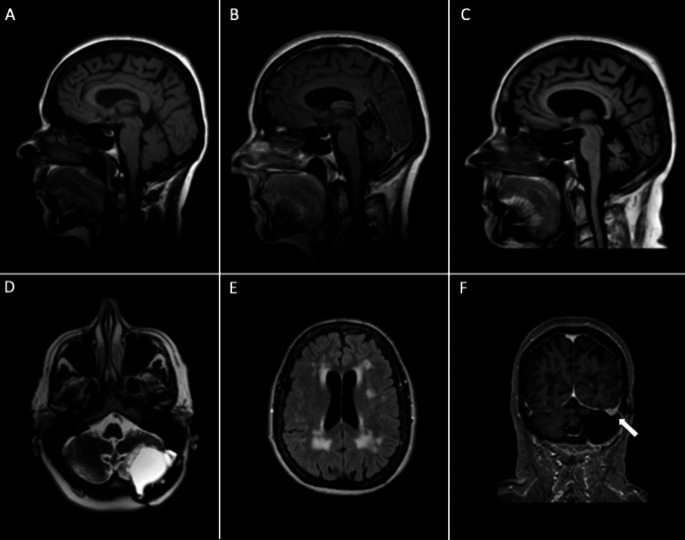
Sagittal T1-weighted brain images showed no cerebellar atrophy at age 54 (one year before surgery) (**A**); mild cerebellar atrophy, predominantly affecting the anterior lobe and superior part of the posterior lobe of the vermis, was observed approximately 6 months post-surgery (**B**); by age 71, moderate diffuse cerebellar atrophy was observed (**C**). Axial T2-weighted image shows the resection cavity on the dorsolateral side of the left cerebellar hemisphere with adjacent gliosis in the left cerebellar hemisphere (**D**), and confluent supratentorial periventricular white matter abnormalities on T2 FLAIR (**E**). Coronal T1 after administration of gadolinium contrast shows a small and stable residual meningeoma (**F**)

At age 67, twelve years post-resection, she developed progressive symptoms of diplopia, speech difficulties, vertigo, and worsening of gait imbalance in a timeframe of four months. Neurological examination showed dysarthria, horizontal diplopia on left lateral gaze, and an impaired tandem gait, but no nystagmus or appendicular ataxia. Brain MRI revealed a small and stable residual cerebellar meningioma on the left side, a surgical resection cavity on the dorsolateral side of the left cerebellar hemisphere with adjacent gliosis in left cerebellar hemisphere extending into the vermis, extensive non-specific supratentorial periventricular white matter abnormalities (WMA) (Fig. 1), and atrophy of the anterior lobe and superior part of the posterior lobe of the cerebellar vermis. A working diagnosis of re-emergence of her cerebellar symptoms, related to the cerebellar lesion and due to a failure of compensatory mechanisms, was considered, and she was referred for rehabilitation.

In the following years, her gait and imbalance worsened, leading to dependency on assistance for walking. Neurological examination at age 71 showed a dysarthria, downbeat nystagmus (DBN) on left and right lateral gaze with rotatory nystagmus, impaired coordination of the limbs most severe on the left side, and gait imbalance. She was not able to walk unaided. Further family history revealed that her patient’s mother experienced symptoms of dizziness, gait imbalance, and speech difficulties at advanced age, without a clear diagnosis. Brain MRI at that time showed the resection cavity with a stable residual meningioma and slight progression of known periventricular WMA. The vermian atrophy remained unchanged. Vestibulonystagmography confirmed a DBN without supporting evidence of vestibular dysfunction. Blood test ruled out vitamin deficiencies, celiac disease, and Wilson’s disease. Initial genetic testing excluded expansions in SCA1-2-3-6-7-17 and dentatorubral-pallidoluysian atrophy, and exome sequencing targeting movement disorders genes was negative. Subsequently, we then requested genetic screening for *FGF14* and *RFC1* repeat expansions. The results showed a GAA expansion of more than 300 repeats (GAA_> 300_) in the *FGF14* gene, compatible with a diagnosis of spinocerebellar ataxia 27B (SCA27B, ATX-*FGF14*).

## Discussion

We here describe a SCA27B patient with a proven pathogenic *FGF14* repeat expansion whose symptoms were initially considered as post-surgical sequelae that deteriorated due to a collapse of compensatory mechanisms with advanced age. Her progressive cerebellar symptoms, the presence of DBN, further family details, and cerebellar atrophy extending beyond the lesional area prompted additional genetic testing, which eventually identified a *FGF14* repeat expansion as the major driver of her progressive cerebellar symptoms over the past four years.

The key triad of SCA27B is an age at onset after 45 years, episodic symptoms, and DBN [3]. Non-cerebellar symptoms can include vestibular areflexia and peripheral axonal sensory neuropathy, dysautonomia, spasticity, and parkinsonian features [4]. Full penetrance of the mutation is observed in individuals with GAA_> 300_ expansions, while incomplete penetrance occurs with GAA_250 − 300_ with indications that this threshold might even be lower [1]. Heterozygous *FGF14* repeat expansions are reported to be a frequent cause of LOCA, with frequencies up to 30% in European cohorts, but a lower frequency of approximately 1% in East-Asian cohorts [1, 3, 5, 6].

Oculomotor disturbances are a key component of the SCA27B phenotype, and have been reported in approximately 95% in a French cohort of SCA27B cases, with DBN being the most commonly reported oculomotor sign in up to 66% of their cohort [2, 3]. Although DBN is not exclusive to SCA27B, its presence combined with cerebellar ataxia should trigger testing for this disorder.

Cerebellar atrophy on brain imaging, predominantly affecting the vermian region, is frequently observed in SCA27B patients, although it is not invariably present [1]. Mild diffuse cerebellar atrophy was also observed in our case, along with extensive periventricular and brainstem WMA. Periventricular WMA were reported in 80% of patient with SCA27B, but the WMA in our case are best compatible with small vessel disease. Interestingly, involvement of superior cerebellar peduncles and the decussation within the midbrain is observed in up to 62% of SCA27B cases, which helps differentiating SCA27B from other LOCA subtypes [7]. These midbrain signal changes were absent in our patient.

The presence of post-surgical cerebellar lesions and residual ataxia led to an initial hypothesis of progression of these residual signs upon ageing. Re-emergence of previous deficits (or recrudescence) triggered by toxic or metabolic factors is a well-known phenomenon in stroke patients, although it is generally transient [8]. The subacute deterioration of cerebellar symptoms after a delay of twelve years, which appeared to be non-transient and progressive, plus the DBN and cerebellar atrophy without signs of new acquired causes, prompted us to consider genetic causes. At the time of initial genetic tests, testing for SCA27B was not yet available.

Our case underlines the importance of considering SCA27B in slowly progressive cerebellar ataxia with oculomotor abnormalities, regardless of the presence of a static cerebellar lesion. We recommend prioritizing genetic screening for SCA27B as the first diagnostic step in these patients; if negative, genetic testing should be expanded to what is routinely done for late-onset ataxias.

## Data Availability

No datasets were generated or analysed during the current study.

## Abbreviations

DBN: Downbeat Nystagmus
FGF14: Fibroblast Growth Factor 14
GAA_> 300_: GAA expansion of more than 300 repeats
LOCA: Late-Onset Cerebellar Ataxia
SCA27B: Spinocerebellar Ataxia 27B
WMA: White Matter Abnormalities

## References

[1] Pellerin D, Danzi MC, Wilke C, Renaud M, Fazal S, Dicaire MJ, et al. Deep intronic FGF14 GAA repeat expansion in late-onset cerebellar Ataxia. N Engl J Med. 2023;388(2):128–41. 36516086 10.1056/NEJMoa2207406 PMC10042577

[2] Pellerin D, Danzi MC, Renaud M, Houlden H, Synofzik M, Zuchner S, et al. Spinocerebellar ataxia 27B: a novel, frequent and potentially treatable ataxia. Clin Transl Med. 2024;14(1):e1504. 38279833 10.1002/ctm2.1504 PMC10819088

[3] Mereaux JL, Davoine CS, Pellerin D, Coarelli G, Coutelier M, Ewenczyk C, et al. Clinical and genetic keys to cerebellar ataxia due to FGF14 GAA expansions. EBioMedicine. 2024;99:104931. 38150853 10.1016/j.ebiom.2023.104931 PMC10784672

[4] van de Warrenburg BP, Kamsteeg EJ. The FGF14 gene is a milestone in ataxia genetics. EBioMedicine. 2024;100:104994. 38301484 10.1016/j.ebiom.2024.104994 PMC10844931

[5] Ando M, Higuchi Y, Yuan J, Yoshimura A, Kojima F, Yamanishi Y, et al. Clinical variability associated with intronic FGF14 GAA repeat expansion in Japan. Ann Clin Transl Neurol. 2024;11(1):96–104. 37916889 10.1002/acn3.51936 PMC10791012

[6] Ouyang R, Wan L, Pellerin D, Long Z, Hu J, Jiang Q, et al. The genetic landscape and phenotypic spectrum of GAA-FGF14 ataxia in China: a large cohort study. EBioMedicine. 2024;102:105077. 38513302 10.1016/j.ebiom.2024.105077 PMC10960143

[7] Chen S, Ashton C, Sakalla R, Clement G, Planel S, Bonnet C et al. Neuroradiological findings in GAA-FGF14 ataxia (SCA27B): more than cerebellar atrophy. medRxiv. 2024.02.16.24302945

[8] Topcuoglu MA, Saka E, Silverman SB, Schwamm LH, Singhal AB. Recrudescence of deficits after stroke: clinical and imaging phenotype, triggers, and risk factors. JAMA Neurol. 2017;74(9):1048–55. 28783808 10.1001/jamaneurol.2017.1668 PMC5710180

